# Acute Psychosis as the Initial Presentation of Systemic Lupus Erythematosus Complicated by Posterior Reversible Encephalopathy Syndrome With Hemorrhage: A Case Report

**DOI:** 10.7759/cureus.102238

**Published:** 2026-01-24

**Authors:** Anas E Ahmed, Bothinah M Al Zahib, Aisha R Al-Rashidi, Abdulnasir H Almassloom, Nawaf S Althobaiti

**Affiliations:** 1 Community Medicine, Jazan University, Jazan, SAU; 2 College of Medicine, Arabian Gulf University, Manama, BHR; 3 College of Medicine, Taibah University, Madinah, SAU; 4 College of Medicine, Alfaisal University, Riyadh, SAU; 5 College of Medicine, Taif University, Taif, SAU

**Keywords:** acute psychosis, autoimmune encephalopathy, intracerebral hemorrhage, neuropsychiatric lupus, posterior reversible encephalopathy syndrome, secondary psychosis, systemic lupus erythematosus, vasogenic edema

## Abstract

Acute psychosis may rarely represent the initial presentation of an underlying systemic and potentially life-threatening medical disorder, posing significant diagnostic and management challenges.

We report the case of a young woman with no prior psychiatric history who presented with abrupt-onset psychosis and behavioral disturbance. Early neuroimaging revealed posterior-predominant vasogenic edema with associated intracerebral hemorrhage, consistent with posterior reversible encephalopathy syndrome (PRES). Further evaluation demonstrated hematological abnormalities, renal involvement, and positive autoimmune serology, leading to the diagnosis of previously unrecognized systemic lupus erythematosus (SLE) with neuropsychiatric involvement. The patient was managed with blood pressure control, immunosuppressive therapy, and supportive neurological and psychiatric care, resulting in marked clinical and radiological improvement. This case emphasizes the importance of maintaining a broad differential diagnosis when evaluating acute psychosis, particularly in young patients with atypical features or abnormal neurological findings. Early neuroimaging and comprehensive systemic workup were critical in identifying a reversible neurological syndrome secondary to an autoimmune disease, allowing timely intervention and a favorable outcome.

The report highlights PRES as an important and potentially reversible cause of acute neuropsychiatric symptoms in SLE and underscores the need for multidisciplinary collaboration to optimize diagnosis, treatment, and prognosis in such complex presentations.

## Introduction

Acute psychosis as the initial manifestation of an underlying systemic medical disorder represents a significant diagnostic challenge in emergency and inpatient settings. While primary psychiatric illnesses are common causes of psychotic presentations in young adults, secondary etiologies such as autoimmune, metabolic, infectious, and vascular conditions must be carefully considered, particularly when neuroimaging or laboratory abnormalities are present [1-3]. Posterior reversible encephalopathy syndrome (PRES) is a clinico-radiological entity characterized by acute neurological symptoms, including altered mental status, seizures, visual disturbances, and, less commonly, prominent psychiatric features [1,3]. It is typically associated with acute blood pressure fluctuations, renal dysfunction, cytotoxic therapies, and autoimmune diseases, and is defined radiologically by reversible vasogenic edema predominantly affecting the posterior cerebral regions [3,4].

Systemic lupus erythematosus (SLE) is a chronic multisystem autoimmune disease with heterogeneous clinical manifestations, including neuropsychiatric involvement [1,3]. Neuropsychiatric systemic lupus erythematosus (NPSLE) encompasses a broad spectrum of central and peripheral nervous system manifestations, ranging from cognitive dysfunction and mood disorders to seizures, cerebrovascular disease, and psychosis [2,3]. PRES has increasingly been recognized as a neurological complication of SLE, often related to endothelial dysfunction, immune-mediated vascular injury, and disease activity, and may be further complicated by intracerebral hemorrhage [1-4]. The coexistence of acute psychosis, PRES with hemorrhagic features, and previously undiagnosed SLE is uncommon and underscores the importance of maintaining a high index of suspicion for systemic autoimmune disease in patients presenting with acute neuropsychiatric symptoms.

## Case presentation

A previously healthy young adult woman in her early 20s, with no known psychiatric illness, was brought to the Emergency Department by her family because of abrupt-onset behavioral changes over 48 hours. According to relatives, she had been well until two days before presentation, when she developed insomnia, marked agitation, and progressive disorganized behavior. This was followed by paranoid ideation, incoherent speech, emotional lability, and visual hallucinations. There was no preceding fever, headache, seizure activity, head trauma, or substance use reported. Her past medical history was unremarkable, with no known autoimmune disease, hypertension, renal disease, or prior neurological events. She was not taking any regular medications, including oral contraceptives or illicit drugs. There was no family history of psychiatric disorders, autoimmune disease, or cerebrovascular events. Review of systems, obtained from family members, revealed intermittent joint pains and photosensitivity over the preceding several months, which had not been medically evaluated, but no prior neuropsychiatric symptoms.

On initial examination, the patient was acutely distressed, restless, and uncooperative. Vital signs showed a blood pressure of 158/96 mmHg, heart rate of 112 beats per minute, respiratory rate of 18 breaths per minute, temperature of 36.8°C, and oxygen saturation of 98% on room air. She was alert but disoriented to time and place, with poor attention and impaired judgment. Speech was pressured and tangential. Thought content was notable for persecutory delusions and visual hallucinations. Cranial nerve examination was limited by poor cooperation, but was grossly intact. Motor examination revealed normal tone and power in all four limbs, with no focal weakness. Sensory examination was noncontributory. Reflexes were symmetric, and plantar responses were flexor bilaterally. No meningeal signs were present. General physical examination revealed a faint malar rash sparing the nasolabial folds and mild non-erosive tenderness of the small joints of the hands without swelling. Cardiovascular, respiratory, and abdominal examinations were otherwise unremarkable.

**Figure 1:**
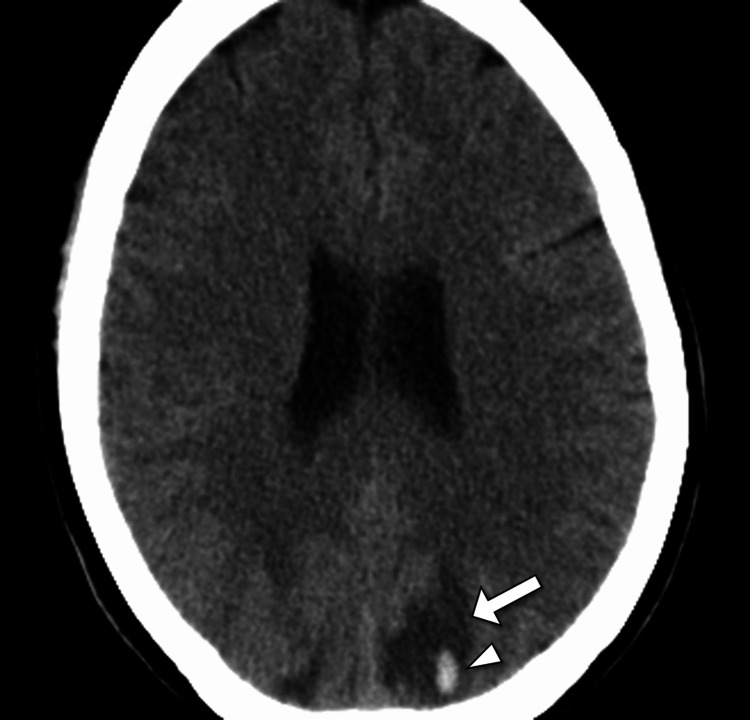
Axial CT image demonstrating subcortical hypodensity and hemorrhage in posterior reversible encephalopathy syndrome Axial non-contrast computed tomography (CT) of the brain shows a subcortical hypodense area in the left parietal lobe (arrow) with an associated focal intraparenchymal hemorrhage (arrowhead). These findings are consistent with posterior reversible encephalopathy syndrome (PRES) with hemorrhagic transformation.

Initial laboratory investigations demonstrated mild normocytic anemia (hemoglobin 10.8 g/dL), leukopenia (white blood cell count 3.2 × 10⁹/L), and thrombocytopenia (platelet count 110 × 10⁹/L). Serum electrolytes, renal function tests, liver enzymes, thyroid function tests, serum ammonia, vitamin B12, and toxicology screen were within normal limits. Inflammatory markers showed an elevated erythrocyte sedimentation rate of 58 mm/hour, with a mildly raised C-reactive protein. Given the acute psychosis with altered mental status, an urgent non-contrast computed tomography (CT) scan of the head was performed, which revealed bilateral parieto-occipital hypodensities consistent with vasogenic edema - more pronounced on the left - along with an acute intraparenchymal hemorrhage in the left occipital lobe, without significant midline shift. These findings raised concern for PRES, complicated by hemorrhage (Figure 1).

**Figure 2:**
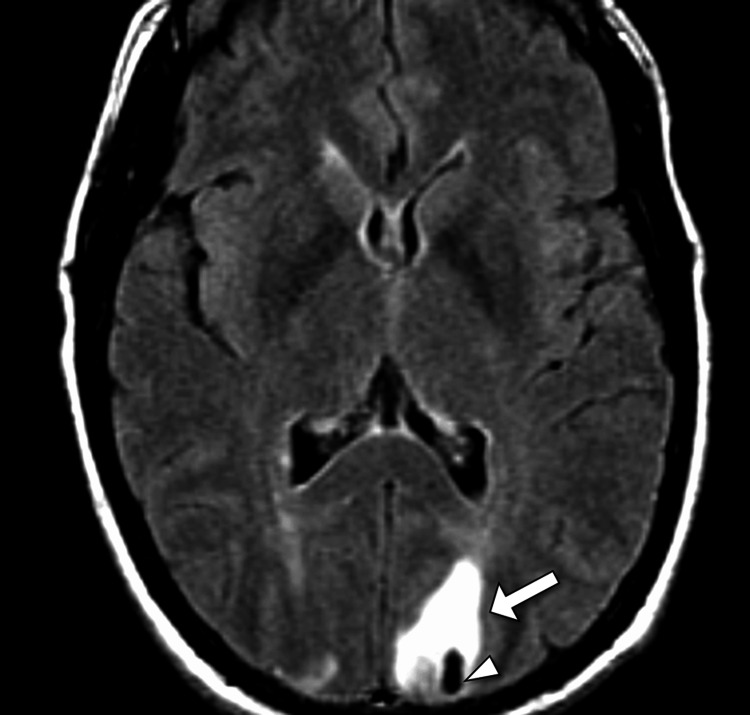
Axial MRI FLAIR showing parietal hyperintensity and hemorrhage in posterior reversible encephalopathy syndrome Axial magnetic resonance imaging (MRI) using fluid-attenuated inversion recovery (FLAIR) demonstrates a hyperintense signal in the left parietal lobe (arrow). A corresponding blooming artifact on susceptibility-weighted imaging (arrowhead) indicates hemorrhage. These features are characteristic of posterior reversible encephalopathy syndrome (PRES) with hemorrhagic involvement.

Subsequent magnetic resonance imaging (MRI) of the brain demonstrated symmetrical hyperintense signals on T2-weighted and fluid-attenuated inversion recovery (FLAIR) sequences involving the bilateral occipital and parietal lobes, extending to the posterior temporal regions, consistent with vasogenic edema (Figure 2). Electroencephalography (EEG) performed due to fluctuating mental status showed diffuse slowing without epileptiform discharges.

Given the radiological diagnosis of PRES in a young patient without chronic hypertension, further etiological workup was undertaken (Table 1). Autoimmune testing revealed a strongly positive antinuclear antibody (ANA) with a homogenous pattern, elevated anti-double-stranded DNA (anti-dsDNA) antibodies, and low complement levels (C3 and C4). Urinalysis demonstrated proteinuria and microscopic hematuria, with a urine protein-to-creatinine ratio of 1.8 g/g. Additional testing showed positive anti-Smith antibodies, while antiphospholipid antibodies (aPL) were negative. Cerebrospinal fluid (CSF) analysis was deferred due to the presence of an intracerebral hemorrhage. Based on the Systemic Lupus International Collaborating Clinics (SLICC) criteria, a diagnosis of SLE, with neuropsychiatric involvement presenting as PRES, was established.

**Table 1:** Summary of laboratory investigations at presentation and during diagnostic workup This table summarizes the hematological, biochemical, immunological, and urinary laboratory findings obtained during the patient’s initial evaluation for acute psychosis and neurological deterioration. Abnormal results supported the diagnosis of an underlying systemic autoimmune disorder with neuropsychiatric involvement. Reference ranges correspond to standard adult values. HPF, high-power field

Parameter	Patient Value	Unit	Reference Range
Hemoglobin	10.8	g/dL	12.0–16.0
White blood cell count	3.2	×10⁹/L	4.0–11.0
Neutrophils	58	%	40–75
Lymphocytes	32	%	20–45
Platelet count	110	×10⁹/L	150–400
Mean corpuscular volume	88	fL	80–100
Erythrocyte sedimentation rate	58	mm/hour	<20
C-reactive protein	12	mg/L	<5
Serum creatinine	0.9	mg/dL	0.6–1.1
Blood urea nitrogen	14	mg/dL	7–20
Sodium	138	mmol/L	135–145
Potassium	4.1	mmol/L	3.5–5.1
Chloride	102	mmol/L	98–107
Bicarbonate	24	mmol/L	22–28
Aspartate aminotransferase	28	U/L	10–40
Alanine aminotransferase	32	U/L	7–56
Alkaline phosphatase	84	U/L	44–147
Total bilirubin	0.7	mg/dL	0.2–1.2
Thyroid-stimulating hormone	2.1	µIU/mL	0.4–4.0
Vitamin B12	420	pg/mL	200–900
Serum ammonia	26	µmol/L	15–45
Antinuclear antibody	Positive	-	Negative
Anti-double-stranded DNA antibody	180	IU/mL	<30
Anti-Smith antibody	Positive	-	Negative
Complement C3	48	mg/dL	90–180
Complement C4	6	mg/dL	10–40
Antiphospholipid antibodies	Negative	-	Negative
Urine protein-to-creatinine ratio	1.8	g/g	<0.2
Urine red blood cells	15–20	/HPF	0–3
Urine white blood cells	2–4	/HPF	0–5
Urine toxicology	Negative	-	Negative

The patient was admitted to the Intensive Care Unit (ICU) for close neurological monitoring. Blood pressure was carefully controlled with intravenous antihypertensive agents to maintain systolic pressures below 140 mmHg. High-dose intravenous methylprednisolone was initiated for suspected lupus-related neuroinflammation, followed by a planned taper. Antipsychotic medication was used short-term to manage severe agitation, and seizure prophylaxis was administered, given the hemorrhagic lesion and EEG findings. Neurosurgical consultation recommended conservative management of the occipital hemorrhage. Over the ensuing days, the patient’s mental status gradually improved, with resolution of hallucinations and normalization of behavior.

She was discharged after three weeks on oral corticosteroids, hydroxychloroquine, and antihypertensive therapy, with close follow-up arranged with rheumatology, neurology, and psychiatry. At six-week outpatient follow-up, she was fully oriented, without residual psychotic symptoms or focal neurological deficits.

## Discussion

The present case illustrates an uncommon but clinically significant presentation of SLE, in which acute psychosis was the dominant initial manifestation and was ultimately attributed to PRES complicated by intracerebral hemorrhage. This constellation of findings underscores the diagnostic complexity of acute neuropsychiatric presentations and highlights the critical importance of considering secondary, potentially reversible causes of psychosis, particularly in young patients without a prior psychiatric history. The case also contributes to the growing body of literature recognizing PRES as an important, albeit underappreciated, manifestation of NPSLE.

Neuropsychiatric involvement in SLE encompasses a broad and heterogeneous spectrum, ranging from mild cognitive dysfunction and mood disorders to severe manifestations such as seizures, cerebrovascular disease, and psychosis [1-4]. Psychosis occurs in a minority of patients with SLE but is a well-recognized and potentially severe manifestation, often associated with high disease activity and serological abnormalities, including elevated anti-dsDNA antibodies and hypocomplementemia, as observed in this case [2,5]. Distinguishing primary lupus psychosis from secondary neuropsychiatric complications such as PRES, infection, metabolic derangements, or medication-related effects is essential, as management strategies and prognoses differ significantly [4-7]. In this patient, the neuroimaging findings of posterior-predominant vasogenic edema with hemorrhagic transformation, along with rapid radiological reversibility following treatment, were more consistent with PRES rather than isolated lupus psychosis or cerebral vasculitis.

The occurrence of intracerebral hemorrhage in PRES, although relatively uncommon, has been increasingly reported, particularly in patients with autoimmune diseases [1-3]. Hemorrhagic complications may result from severe endothelial injury, reperfusion injury, or coexisting thrombocytopenia and coagulopathy, all of which are relevant in SLE. The presence of hemorrhage can complicate both diagnosis and management, limiting the use of certain diagnostic procedures such as lumbar puncture and increasing the risk of neurological morbidity [2,6]. Nevertheless, conservative management with close monitoring, blood pressure control, and treatment of the underlying cause is often sufficient, as demonstrated by the favorable outcome in this case.

From a diagnostic perspective, this case emphasizes the pivotal role of early neuroimaging in patients presenting with acute psychosis and altered mental status. Reliance solely on clinical psychiatric assessment risks misdiagnosis and delays in identifying potentially life-threatening neurological conditions [2-5]. CT remains a valuable initial modality in the emergency setting, particularly for detecting hemorrhage, while MRI provides superior characterization of PRES and helps differentiate it from ischemic stroke, infection, demyelination, or vasculitis [1,6]. Additionally, a systematic laboratory evaluation, including autoimmune serology, is essential in young patients with unexplained neuropsychiatric symptoms, especially when accompanied by systemic features such as cytopenias, rash, or renal abnormalities [2-5].

Therapeutically, management of PRES centers on prompt identification and correction of precipitating factors [1,4]. In SLE-associated PRES, this includes aggressive control of blood pressure, withdrawal of offending agents, if present, and treatment of active lupus with immunosuppressive therapy. High-dose corticosteroids are commonly employed in the setting of NPSLE, and were associated with marked clinical improvement in this patient. The favorable neurological and radiological recovery observed reinforces the concept that PRES is potentially reversible when recognized early and managed appropriately. However, delayed diagnosis, or inadequate treatment, may result in permanent neurological deficits or death, particularly in cases complicated by hemorrhage or infarction [5-8].

## Conclusions

This case highlights the importance of a comprehensive and multidisciplinary approach to patients presenting with acute psychosis, particularly when clinical features are atypical for a primary psychiatric disorder. The identification of PRES with associated intracerebral hemorrhage on early neuroimaging was pivotal in prompting an expanded diagnostic evaluation that ultimately revealed previously undiagnosed SLE with neuropsychiatric involvement. Prompt recognition of the underlying autoimmune etiology allowed timely initiation of immunosuppressive therapy and strict blood pressure control, leading to significant clinical and radiological recovery.

The case underscores that acute psychosis may be the initial manifestation of serious systemic and neurological disease, and emphasizes the critical role of neuroimaging, autoimmune workup, and close interdisciplinary collaboration in such presentations. Early diagnosis and targeted management are essential to prevent irreversible neurological injury and to improve outcomes, reinforcing the need for clinicians to maintain a high index of suspicion for secondary causes of psychosis, including autoimmune disorders, in acute and atypical neuropsychiatric presentations.
